# Optimization of enzymatic fragmentation is crucial to maximize genome coverage: a comparison of library preparation methods for Illumina sequencing

**DOI:** 10.1186/s12864-022-08316-y

**Published:** 2022-02-01

**Authors:** Teodora Ribarska, Pål Marius Bjørnstad, Arvind Y. M. Sundaram, Gregor D. Gilfillan

**Affiliations:** Department Medical Genetics, Oslo University Hospital and University of Oslo, Kirkeveien 166, 0450 Oslo, Norway

**Keywords:** Whole genome sequencing, library preparation, enzymatic fragmentation, insert size, Next Generation Sequencing

## Abstract

**Background:**

Novel commercial kits for whole genome library preparation for next-generation sequencing on Illumina platforms promise shorter workflows, lower inputs and cost savings. Time savings are achieved by employing enzymatic DNA fragmentation and by combining end-repair and tailing reactions. Fewer cleanup steps also allow greater DNA input flexibility (1 ng-1 μg), PCR-free options from 100 ng DNA, and lower price as compared to the well-established sonication and tagmentation-based DNA library preparation kits.

**Results:**

We compared the performance of four enzymatic fragmentation-based DNA library preparation kits (from New England Biolabs, Roche, Swift Biosciences and Quantabio) to a tagmentation-based kit (Illumina) using low input DNA amounts (10 ng) and PCR-free reactions with 100 ng DNA. With four technical replicates of each input amount and kit, we compared the kits’ fragmentation sequence-bias as well as performance parameters such as sequence coverage and the clinically relevant detection of single nucleotide and indel variants. While all kits produced high quality sequence data and demonstrated similar performance, several enzymatic fragmentation methods produced library insert sizes which deviated from those intended. Libraries with longer insert lengths performed better in terms of coverage, SNV and indel detection. Lower performance of shorter-insert libraries could be explained by loss of sequence coverage to overlapping paired-end reads, exacerbated by the preferential sequencing of shorter fragments on Illumina sequencers. We also observed that libraries prepared with minimal or no PCR performed best with regard to indel detection.

**Conclusions:**

The enzymatic fragmentation-based DNA library preparation kits from NEB, Roche, Swift and Quantabio are good alternatives to the tagmentation based Nextera DNA flex kit from Illumina, offering reproducible results using flexible DNA inputs, quick workflows and lower prices. Libraries with insert DNA fragments longer than the cumulative sum of both read lengths avoid read overlap, thus produce more informative data that leads to strongly improved genome coverage and consequently also increased sensitivity and precision of SNP and indel detection. In order to best utilize such enzymatic fragmentation reagents, researchers should be prepared to invest time to optimize fragmentation conditions for their particular samples.

**Supplementary Information:**

The online version contains supplementary material available at 10.1186/s12864-022-08316-y.

## Background

During the last decade, Illumina technology has come to dominate short read next generation sequencing (NGS), offering cost-effective high precision data for a wide variety of applications such as whole genome sequencing (WGS), metagenomics and transcriptomics. In medical genetics, WGS is increasingly applied to identify disease-causing genetic variation (SNPs or structural variants), disease susceptibility, cancer evolution and drug response, among a plethora of other applications [1–3].

Since the cost of next generation sequencing is still high and often the amount of available DNA from the biological source is limited, methodological efforts are constantly underway to improve the efficiency of WGS, i.e. extracting the most unique genetic information, with the highest possible quality and coverage, from a variety of input DNA amounts and qualities, at the lowest cost and shortest hands-on time.

Library preparation is an essential process preceding sequencing itself, and comprises several aspects that affect the efficiency of WGS. It typically involves the following main steps: fragmentation of the input DNA, end-repair and A-tailing of the DNA fragments, ligation of indexed sequencing adapters and optional amplification of the ligated products. In addition, one or more cleanup steps are necessary in between steps to purify the DNA reaction products of reagents from the previous reaction.

The most commonly used methods for fragmentation of genomic DNA are sonication, tagmentation (i.e., transposition of partial adapters into the DNA), and enzymatic digestion by DNA endonucleases. Prior to the widespread adoption of enzymatic fragmentation, sonication was preferred, as it produces near-random fragmentation, and fragment length can be adjusted by varying sonication time and strength. However, this requires a DNA sonication instrument and in some cases also special consumable sonication tubes, adding considerable cost and handling time to the procedure. Based on sonication, Illumina’s Truseq PCR-free library preparation reagents were the first commercial kit enabling PCR-free library preparation for Illumina sequencers. However, this kit requires DNA input of 1 microgram DNA, which is in many cases not available.

Greater flexibility of input amount and a significantly quicker protocol was subsequently offered by Illumina’s tagmentation based Nextera reagents, albeit not allowing PCR-free prep (until recently, in its renamed version Illumina DNA prep). The principle of tagmentation is the insertion by transposition of partial sequencing adapter sequences in genomic DNA that in effect fragments and adds adapters in a single step. Subsequently, the adapters are extended to full length by PCR (in the Nextera DNA flex kit) and through an undisclosed PCR-free method in the Illumina DNA PCR-free kit. The length of the DNA between the transposed adapters is dependent on the size of the beads and the concentration of the transposomes (transposase loaded with adapters) coating on them [4], which is fixed for the respective kit. The sole possibility to modulate this length is by means of size selection after library preparation is complete, which may discard a considerable portion of the library.

Endonuclease-based fragmentation for NGS was initially feared to suffer from enzyme cut-site preference bias and the introduction of artefacts [5]. However, recent commercial enzyme preparations have largely alleviated these concerns [6], and several competing library prep kits that use enzymatic fragmentation have emerged. Offering quick and simple workflows, high flexibility of DNA input amounts, PCR-free options with approximately 100 ng DNA, and importantly a lower price, these kits offer attractive alternatives to sonication and tagmentation. Here, we compare the performance of several of these kits.

In order to evaluate the performance and sequencing data quality produced with enzymatic fragmentation-based library prep kits, we performed WGS using four such kits (from New England Biolabs, Quantabio, Swift Biosciences and Roche) and the Nextera DNA flex tagmentation based kit (from Illumina) with 10 and 100 ng DNA inputs, and sequenced them on an Illumina HiSeq X instrument. All of the tested kits reproducibly delivered similar high-quality data in terms of coverage and precision of single nucleotide variant (SNV) and indel detection. We observed that libraries with DNA insert size longer than the combined sequencing reads length exhibited improved performance than those with shorter length. However, there is an optimum insert length, beyond which further increase in insert length does not augment the sequencing data performance but can reduce clustering efficiency and data yields.

## Results

### Study design

We compared the WGS performance of four enzymatic fragmentation-based library preparation kits: NEBNext Ultra II FS from NEB (hereafter referred to as NEB), Swift 2S Turbo flexible from Swift Biosciences (hereafter Swift2S), SparQ DNA Frag and Library Prep from Quantabio (Quanta) and KAPA HyperPlus from Roche (Kapa) with the tagmentation-based Nextera DNA FLEX kit from Illumina (Nextera). In our study design we prepared libraries from 10 ng and 100 ng input DNA amounts, whereby the 100 ng input reactions were PCR-free (or with minimal PCR cycles, where absolutely required; see Table 1 for details). An entirely PCR-free option was not possible for Nextera and NEB kits, in which PCR is necessary to complete the sequencing adapters (and add indexes). With four technical replicates for each input amount and kit, we aimed to test the reproducibility and robustness of the kits, with respect to fragment size distribution and quality of the sequencing data. As input DNA we used genomic DNA from the human fibroblast cell line NA12878 (purchased from Coriell Institute) that has been well characterized (e.g. for indels and single nucleotide variants) and often used as standard control DNA source for genomic studies- called therefore also “genome in a bottle” [7, 8]. Library concentrations produced by each replicate are summarized in Additional Table 1. The libraries from 10 and 100 ng DNA inputs, each in four technical replicates, were pooled and sequenced over 20 lanes of HiSeq X flowcells (i.e. four lanes per kit), with 150 bp paired end reads.

**Table 1:** Mean DNA insert sizes upon fragmentation and after sequencing achieved with different library preparation kits

Kit	Target insert size [bp]	10 ng input				100 ng input			
		frag. time [min]	Insert size [bp]		PCR cycles	frag. time [min]	Insert size [bp]		PCR cycles
			by Tapestation	by seq. Reads			by Tapestation	by seq. Reads	
Nextera DNA flex (Illumina)	450	15*	418(±5)	326(±2)	8	15*	479(±2)	366(±2)	5
Kapa HyperPlus (Roche)	350	20	345(±7)	240(±9)	9	20	-	227(±3)	-
SparQ Fragment and Library Prep (Quantabio)	350	16	245(±8)	185(±3)	9	10	-	244(±10)	-
Swift 2S turbo flexible (Swift)	350	10	422(±9)	330(±12)	6	8	-	226(±7)	-
NEBNext Ultra II FS DNA library prep (NEB)	200-450	15	303(±9)	206(±7)	7	15	276(±7)	188(±6)	3

**Fig. 1:**
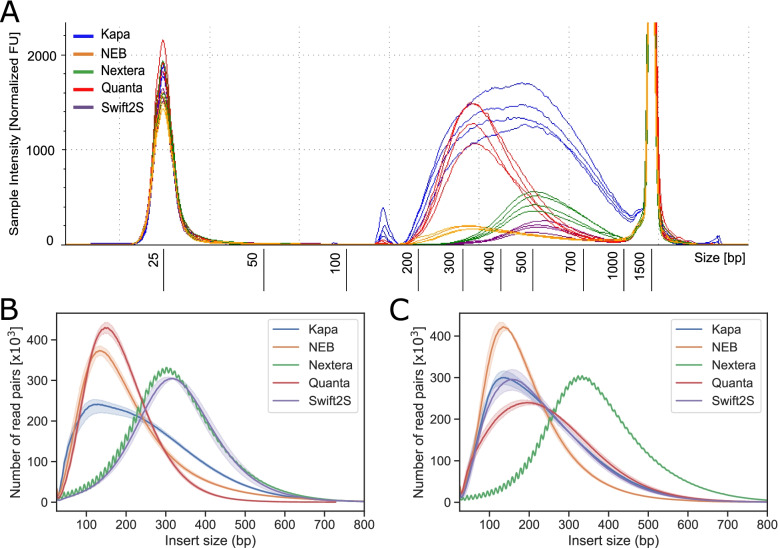
DNA insert size assessment of libraries prepared with enzymatic fragmentation and tagmentation. **A** Tapestation D1000 electorphoresis profiles for libraries prepared with 10 ng input DNA in four technical replicates each with Nextera DNA flex (Illumina), NEBNext Ultra II FS (NEB), Kapa Hyper Plus (Roche), SparQ DNA library (Quantabio) and Swift2S turbo flex (Swift) kits. DNA insert size of libraries was assessed from the sequencing reads of (**B**) libraries from 10 ng and (**C**) 100 ng DNA input after trimming of adapter sequences. The shaded regions are 95 % confidence intervals of the distributions sampled by the replicates (The band is hardly visible for Nextera, because the replicates are very similar)

Libraries were prepared from 10 and 100 ng of human NA12878 DNA using enzymatic fragmentation and tagmentation (*) based library prep kits, employing the given fragmentation times and PCR cycles. Bead-based purifications were performed according to the individual instructions of the DNA library preparation kits. Insert DNA sizes were calculated by subtracting the adapter length from the mean fragment size based on Tapestation D1000 profiles and from the sequencing reads upon mapping after trimming of adapters, standard deviation is given in brackets. PCR-free libraries do not migrate properly on Tapestation, due to forked adapters, therefore there is no insert size data based on Tapestation. For additional information, see also Fig. 1 and Additional Fig. 1A

### Fragmentation

The enzymatic fragmentation-based protocols require different fragmentation conditions (time and temperature) depending on the input DNA amount and the desired insert fragment length. In each case, we used the manufacturer’s recommended conditions respectively for 10 and 100 ng DNA input whilst aiming for average 350 bp insert DNA size (excluding the adapters; see Table 1). The fragment distribution of the final libraries was assessed by capillary electrophoresis (Fig. 1A and Additional Fig. 1A) and in addition calculated from the mapped paired end sequencing reads (Fig. 1B and C, Table 1). Gel electrophoresis was not performed on PCR-free libraries, as their termini are partially single stranded (with Y-shaped or also called forked adapters) which affects migration by gel electrophoresis. Therefore, electrophoresis insert-sizes have been omitted for the PCR-free libraries produced from 100 ng DNA with the Kapa, Quanta and Swift2S kits.

All kits except Kapa produced a relatively narrow distribution of fragment sizes (Fig. 1A). The fragment distributions of Quanta and NEB libraries exhibited a notable skewing towards inserts shorter than the median fragment length. Nonetheless, based on the gel migration profiles for 10 ng input libraries, all of the kits efficiently and reproducibly fragmented the DNA using the recommended settings. The average insert size was close to the 350 bp target length for Kapa and NEB libraries and 450 bp for Nextera, while Quanta and Swift2S libraries produced considerably shorter (245 bp) or longer (422 ng) average lengths respectively (summarized in Table 1). Note that we did not optimize experimentally the fragmentation conditions, as recommended in the manuals, but rather used directly the suggested settings.

We also measured the insert sizes of the libraries obtained from the paired end sequencing reads (Fig. 1B and C, Table 1). Insert sizes observed by sequencing reflected the diverse sizes seen by electrophoresis, i.e., kits that had produced longer inserts observed by electrophoresis, also showed longer inserts as measured by the distance between paired sequencing reads. Among all kits, the insert lengths varied from 185 to 366 bp, whereby Quanta and NEB libraries exhibited shorter insert sizes (in the range of 185-227 bp) than Swift2S and Nextera libraries (in the range of 326-366 bp), while KAPA libraries were in the middle range (227-240 bp). The mean insert sizes were not significantly different for Nextera and Swift2S at 10 ng inputs, and for Kapa and Swift2S at 100 ng inputs, but for all other pairwise comparisons, there was a significant difference between the mean insert sizes (p < 0.04). We consistently observed that the mean insert-sizes measured between sequencing reads were considerably shorter (by ca. 60-100 bp) than lengths estimated by Tapestation (Table 1). The kits yielded reproducible fragment size distributions, such that the mean insert sizes differ by only about 10 bp between replicates (Table 1). The jagged periodicity of the Nextera insert size profiles has been observed before and can be explained by steric hindrance between adjacent transposases influenced by the helical pitch of DNA, which is ca. 10 bp long [9].

### Sequence bias

To evaluate possible sequence coverage bias arising from use of the different library preps, we analysed the GC content of the mapped reads, but found this to be very similar for all kits. Overall, the differences observed were minor, being less than 2-fold deviation from expected GC-content across the 20-70% GC spectrum analysed (Additional Fig. 1B-E). The greatest differences were observed in libraries from 10 ng DNA input. A closer look at these differences, represented by the observed vs. expected GC content of the whole reads, shows that Nextera and Quant libraries maintained the closest relationship between observed:expected GC content. Swift2S libraries exhibited a greater proportion of reads with lower than the expected 40% GC content, while Kapa and NEB libraries showed a slight bias towards high GC content (Additional Fig. 1D).

To determine whether enzymatic fragmentation is prone to sequence specific nuclease bias, we analysed the sequence composition at the beginning of the reads, corresponding to the endonuclease cut site. All enzymatic nuclease-based kits exhibit a similar sequence pattern in the first 10 bases, having a higher proportion of A and T, being followed by an even base distribution corresponding to the expected 60 % AT and 40 % GC base content (Additional Fig. 1F). The Nextera kit exhibited TA-rich sequence bias at the beginning of the reads as has been previously observed [10], that is slightly higher than the bias of the enzymatic fragmentation preps (Additional Fig. 1F), also visible as a spike at low observed:expected GC ratio (Additional Fig. 1E).

### Sequencing performance of library prep kits

Sequencing yielded a variable number of reads; ca. 100-300 million reads per library (Additional Fig. 2A). The PCR-free libraries prepared with KAPA and Quanta kits from 100 ng input DNA yielded about three times fewer reads than their 10 ng input counterparts prepared with PCR amplification (Additional Fig. 2A). These observations can likely be attributed to imprecise quantification of the libraries (as the size of PCR-free libraries cannot be correctly determined by electrophoresis), but could also be due to lower clustering efficiency of PCR-free libraries, which can contain incomplete molecules having only a single or no adapter ligated.

All libraries exhibited low duplication rates after excluding duplicates produced during Illumina’s ExAmp™ amplification (Additional Fig. 2B). The duplication rates were below 3% for PCR-free libraries and as expected were slightly higher for PCR-amplified libraries, in the range of 3-6% (Additional Fig. 2B). In the absence of PCR amplification, “duplicate” reads originate from true biological duplicates (DNA fragments with the same start and end), which are expected due to the sequence specificity of enzymatic DNA digestion and tagmentation, in addition to the high sequencing coverage. The base quality of all libraries was similarly high (> 80%; Additional Fig. 2C), enabling their comparison.

**Fig. 2:**
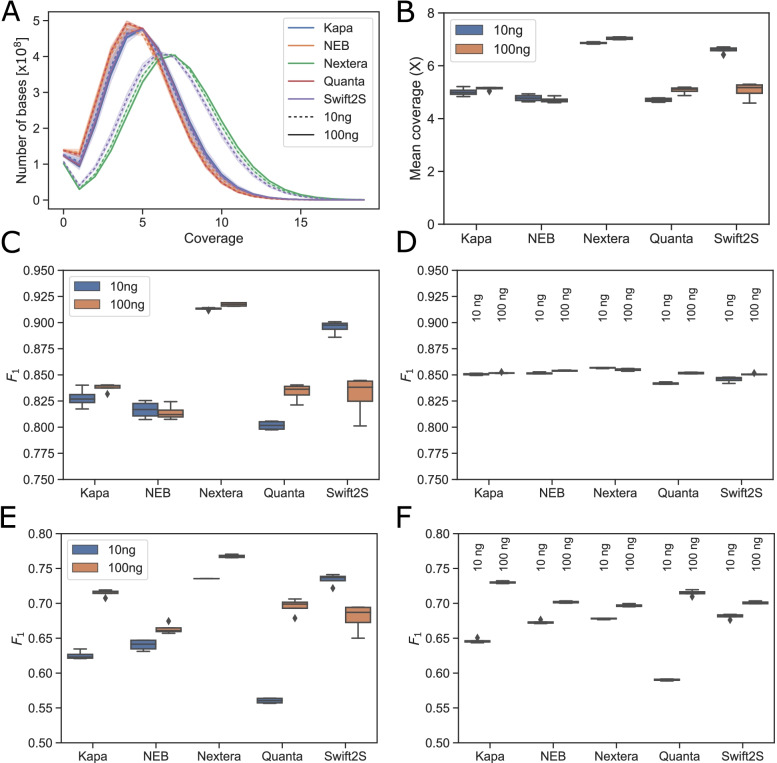
Performance of sequencing data produced with different WGS library preparation kits. Human genome coverage from 90 million reads from libraries from 10 ng and 100 ng input DNA in four technical replicates represented as distribution (**A**) or as average value (**B**). Precision score (F1) for SNP detection (**C**) and indel calling **E**) using 90 million reads, or using a fixed coverage of 5.4x - respectively (**D)** and (**F**)

Since the number of reads output by the sequencer is influenced strongly by the loading concentration, (which is affected by variations in pipetting, QC practices, and library insert size), to avoid any undue bias we used the same number of reads for the functional data analyses (genome coverage, SNV and indel detection) for each kit, by randomly down-sampling to 90-million read pairs per replicate. We then computed the mean coverage of the human genome (Fig. 2A and B). Nextera (both 10 and 100 ng inputs) and Swift2S (10 ng input only) exhibited considerably higher depths than the other kits (Fig. 2A and B) with significance p < 1e-4. This closely mirrors the fragment and insert sizes of the libraries, whereby Nextera and Swift2S turbo 10 ng libraries had the longest insert sizes (ca. 330 bp), while at 100 ng input DNA, only Nextera exhibited an average insert size above 300 bp (namely 366 bp), (Fig. 1, Table 1).

The observed lower coverage per read for libraries with short inserts can be explained with the difference between the insert DNA length and the cumulative length of the sequencing reads (2x150 bp=300 bp). An insert shorter than 300 bp will result in overlap of the 150 bp sequencing reads. This results in redundant sequencing data that reduces the effective overall genome coverage, but nonetheless contributes to sequencing depth.

The distribution of coverage depth per genomic locus should ideally follow a Poisson distribution if the reads are randomly distributed across the genome [11]. High-throughput sequencing data are known to deviate from this ideal [12], but the extent of the disagreement can be used to estimate biases in the coverage distribution. The coverage histograms were fitted with Poisson distributions (Additional Fig. 3A). We expected deviation at zero coverage compared to the Poisson distribution, because the reference genome contains some regions that are difficult to cover with short-read technology, as well as the Y chromosome, which should not be present in NA12878. The number of nucleotide positions without coverage over the expected quantity from the Poisson model, as a percentage of the genome size, is between 3.4 and 4.1 % for the different kits (Additional Fig. 3B). The longer-insert libraries (Nextera at both 10 and 100 ng, and Swift2S at 10 ng) left a smaller percentage of the genome without coverage compared to the other kits (*p* < 1e-6).

To evaluate how the sequencing data from each library prep performs in terms of ability to detect true genomic SNP and insertion-deletion (indel) variants while avoiding false positives, we compared the calls to a database of known true variants in the NA12878 cell line. The recall is defined as the fraction of known true variants that are called, and the precision is the fraction of the called variants that are actually true. To find a single number per library representing the performance, we computed the F_1_ scores, defined as the harmonic mean of the precision and recall of variant detection [13]. Compared to the arithmetic mean, the harmonic mean puts more emphasis on the smaller of its arguments. For this purpose, we used either random samples of 90 million sequencing read pairs or a normalized high quality coverage depth of 5.4x for each library. The SNP calling performance was highest for the libraries with longer inserts using the fixed input read number (Fig. 2C), while this trend disappeared if we used a fixed coverage depth as input (Fig. 2D). Correlating the individual library insert sizes with F_1_ scores revealed a strong relationship between insert size and SNP detection with a fixed read number, which was lost if correlated to fixed coverage depth (Additional Fig. 4A and B). At a fixed read number, the higher coverage in longer-insert libraries therefore allows better SNP detection.

Longer insert libraries also performed slightly better at indel calling, at a fixed read number, but with a less clear trend (Fig. 2E). We observed a similar correlation between F_1_ score and insert size for indel calling (Additional Fig. 4C and D). In this case, the correlation was weaker than for SNP-calling (*r*^2^ = 0.66, versus *r*^2^ = 0.93 for SNPs) indicating that the performance is driven by other variables than just insert size. However, when comparing library performance using fixed-coverage data (Fig. 2F), 100 ng input libraries consistently performed better than 10 ng input libraries, suggesting that indel calling performance can be negatively affected by PCR amplification. PCR amplification has been reported to introduce a number of error-prone indels into the library, and therefore the PCR cycles should be kept to a minimum [14].

We also analysed the intersections of variant calls, shown in Additional Fig. 5. If specific kinds of variants are preferentially called by some of the kits, due to biases, we might expect to see a larger overlap of calls for those kits. The results show that approximately 40 % of the true positive SNPs and 20 % of the true positive indels were called by all kits. This low level of sensitivity is not surprising given the low (5.4x) coverage. Nonetheless, the data indicated that kit performance was generally similar.

## Discussion

In this study we examined the performance of four WGS library preparation kits that employ enzymatic DNA fragmentation in comparison to the “tagmentation”-based Illumina DNA library preparation kit. We evaluated the reproducibility of fragmentation, sequence bias, quality and coverage of the sequencing as well as the combined precision and recall (F1 score) for SNV and indel detection.

Our results show that the four tested enzymatic fragmentation-based library preparation kits produce WGS libraries with reproducible DNA insert sizes, but require experimental optimization of the fragmentation conditions in order to achieve the optimal sequencing insert size. While we have not tested different conditions, the amounts and sources of DNA and elution buffers are factors that will likely affect the fragmentation, and may need optimization on a per-case basis. In contrast, the need for initial optimization is largely avoided when using bead-linked tagmentation, as in the Nextera flex kit. With this method, the degree of fragmentation is limited by the size of the bead, allowing a saturating amount of input DNA to bind and be “tagmented” by the anchored transposases. As such, the average fragment length can only be modulated by size selection following completion of the library prep. According to the manufacturer, the fragmentation profile and library output should be constant for inputs above 100 ng that saturate the transposase on the beads [4]. We observed slightly shorter insert sizes from lower inputs suggesting that initial optimization is advisable also with this kit when using low inputs.

We consistently observed that the mean insert-sizes measured between sequencing reads were considerably shorter (by ca. 60-100 bp) than lengths estimated by Tapestation. This phenomenon is based on the fact that shorter sequences cluster more efficiently than longer fragments on the patterned flow cell during exclusion amplification [15]. This effect was smallest for Quanta libraries, likely because their average size before sequencing was shortest and most optimal in terms of clustering efficiency, and largest with Nextera libraries, which exhibited the highest proportion of long fragments.

The enzymatic nuclease-based kits exhibited a similar sequence insertion bias as indicated by a similar sequence pattern in the first 10 bases of each read. This suggests that they employ a similar mix of nucleases. The tagmentation by Nextera exhibited a different bias, as described previously [16]. There was no clear relationship between observed:expected GC content and number of PCR cycles applied during the preps, nor with insert size, so it would appear that these are intrinsic properties of the enzymes employed in the kits. When examining the observed:expected GC content of the whole reads, the differences between the libraries are minimal, mostly visible in libraries from 10 ng DNA input and can largely be attributed to the low DNA input and PCR.

We obtained different numbers of sequence reads from the libraries, which can be attributed to imprecise quantification and differences in clustering efficiency. Nevertheless, the base calling quality (Q30) was above 80% for all libraries and the duplication rates were comparably low for all. To fairly compare the performance of the libraries in terms of genome coverage and SNV and indel detection, we either considered a fixed number of sequencing reads for each library, or a fixed coverage. All of the tested kits reproducibly delivered similar high-quality data in terms of coverage and precision of single nucleotide variant (SNV) and indel detection. Surprisingly, we observed that libraries with DNA insert size longer than the combined sequencing reads length exhibited improved coverage and variant detection performance than those with shorter length. This is achieved by avoiding overlap of the sequencing reads by libraries and allowing for more unique information to be gathered. When computing the coverage, bases in the overlap region are only counted once for each read *pair*, because they do not give additional unique information as independent fragments. In extreme cases, where the insert is shorter than the read length, further loss of coverage occurs as sequence is wasted as part of the read extends into the library adapter sequence. Since shorter insert fragments cluster and sequence more efficiently on Illumina sequencing instruments, this is of particular concern for libraries with a broad size distribution, or those with a size distribution skewed towards shorter inserts.

We underline that we are not advocating that researchers aim for as long inserts as possible. There is an optimum insert length, beyond which further increase in insert length does not augment the sequencing data performance but can reduce the clustering efficiency and data yields. Once above the threshold where inserts do not result in overlapping paired reads, there are no further expected benefits to genome coverage. On the contrary, over-long libraries are in our experience more difficult to quantify accurately in order to obtain optimal loading and output of Illumina sequencers. They are also at a disadvantage compared to shorter libraries during the clustering on Illumina patterned flow cells, evident by the greatly reduced lengths of the sequenced fragments as comparted to the input fragments. Furthermore, long fragments have been shown to achieve lower base quality, especially in the second read [17].

We also wish to underline that whilst the above conclusions are particularly relevant to whole-genome resequencing of organisms from which high quality DNA is routinely available (e.g. human WGS), alternative applications of WGS may benefit from different choices of insert length. For example, applications that entail low-coverage sequencing such as genome-skimming, may benefit from avoiding a wide range of insert sizes and resulting wider range of genome coverage, as this may in some instances lead to consistent drop-out of low-coverage areas, despite a higher average coverage [18, 19]. Overlapping reads not only increase the accuracy of base-calls, but the resulting longer merged reads can aid in read mapping, particularly if ambiguous base calls are present. Applications that may benefit from the deliberate use of overlapping paired end reads include ancient-DNA and forensic sequencing [20]. In addition, the software and analysis strategy chosen should also be considered on a per-application basis prior to choosing a desired insert length (for example see [21]).

A recent publication detected increased insertion /deletion artefacts in libraries prepared with enzymatic fragmentation relative to those prepared by sonication [22]. Although the authors produced a software tool to detect such artefacts, this finding reinforces the reputation of sonication as the “gold-standard“ method of DNA fragmentation, even though some simple steps must also be taken with sonication to avoid introducing sequencing artefacts [23]. Nonetheless, we speculate that our observation that library insert size affects genome coverage and variant detection will also be valid for libraries produced by sonication.

Optimization of library insert sizes has previously been discussed as a factor in genome assembly [24], or for exome-sequencing applications [25, 26]. Here, we have observed the effect of insert size applied to resequencing of the human genome, but we expect the conclusions to be more widely applicable also to other genomes. Importantly, our results show that independent of the kit, libraries with DNA insert size longer than the sum length of both sequencing reads, yield better sequencing data in terms of coverage and variant detection than libraries with shorter insert lengths by avoiding “loss” of sequencing information due to overlapping sequencing reads.

## Conclusions

Recent developments in commercially-available whole genome library preparation kits for NGS have enabled faster and more streamlined workflows, starting from flexible DNA input amounts and allowing PCR-free library preparation at lower costs. We tested the performance of four WGS library prep kits employing enzymatic fragmentation in comparison to the transposition-based Nextera DNA flex kit. We observed comparable quality of the sequencing results that led to efficient detection of genomic variants, from which we conclude that enzymatic-fragmentation based kits are a good alternative to the tagmentation based Nextera Flex kit.

Independent of kit chosen to prepare WGS libraries, we observed superior genome coverage and indel calling in libraries with average insert sizes longer than the sum of the paired read lengths. In contrast, shorter-insert fragments are preferentially sequenced with Illumina technology. The ideal sequencing library for WGS would therefore consist of a narrowly distributed peak of DNA fragments, the majority of which lie above the sum of the paired read lengths.

Both enzymatic and tagmentation fragment sizes can be refined after library preparation by size selection, although this comes at the expense of library yield, as unwanted fragments are discarded. Compared to the Nextera Flex kit, the enzymatic fragmentation reagents allow greater control of fragment size through variation of fragmentation time. However, despite following manufacturer’s instructions and using a high-quality preparation of reference genomic DNA as input, we observed deviations from the intended insert size with three out of four enzymatic fragmentation kits. It may therefore be necessary to invest more time optimizing the fragmentation conditions in order to reap the cost benefits these kits offer.

## Materials and methods

### Library preparation

Whole genome DNA library preparation was performed using DNA NA12878 as input (Coriell Institute, NJ, USA). For each library preparation kit under study, four replicate libraries were prepared from both 10 ng and 100 ng input DNA. The following library prep kits were used according to the manufacturer’s manuals with the given modifications: Nextera^TM^ DNA flex library prep (Cat. Nr. 20018705, Illumina) using IDT for Illumina – Nextera DNA UD Indexes Set A (Cat. Nr. 20027213, Illumina): kit manual version 1000000025416v00; NEBNext® Ultra^TM^ II FS DNA Library Prep Kit for Illumina® (Cat. Nr. E7645S, New England Biolabs) with NEBNext Multiplex Oligos for Illumina (96 Unique Dual Index Primer Pairs, Cat. Nr. 6440L, New England Biolabs), kit manuals “E7805, E6177 & INPUTS ≤ 100 ng” (as of 04.08.2019); KAPA HyperPlus DNA library prep kit (Cat. No. KK8510, Roche) using IDT for Illumina – TruSeq DNA UD Indexes (Cat. Nr 20020178, Illumina), kit manual version KR1145-v4.17; SparQ DNA Frag and Library Prep kit (Cat. Nr. 95194-024, Quantabio) using IDT for Illumina – TruSeq DNA UD Indexes (Cat. Nr 20020178, Illumina) according to the kit manual (version: 95194/IFU-122.1 REV 01); Swift 2S Turbo flexible DNA library kit (Cat. No. 44024) using IDT for Illumina – TruSeq DNA UD Indexes (Cat. Nr 20020178, Illumina), kit manual version 2.0.

Fragmentation times and amplification cycles were applied according to the ranges recommended in each kit as summarized in Table 1. SparQ PureMag beads (Cat. Nr. 95196-450, Quantabio)-based cleanups used were performed according to the individual instructions of the DNA library preparation kits.

### Quality control, pooling and sequencing of libraries

Quantity and fragment sizes of all libraries were assessed by capillary electrophoresis on a Tapestation 2200 (Agilent) using D1000 Screen Tapes. Libraries were quantified by qPCR using KAPA library quantification kit (Cat. Nr. KK4854 – 07960298001, Roche) using a Roche Lightcycler 480 II real time PCR system. Separate pools were prepared for the libraries of each kit, whereby each pool consisted of 8 equimolar libraries (4 with 10 ng input and 4 with 100 ng input). Each pool was sequenced on four lanes of a HiSeq X (Illumina; RTA v. 2.7.7) with 150 bp paired end reads and demultiplexed into individual fastq datasets using bcl2fastq v. v2.18.0.12.

### Data analysis

Sequence data quality was checked using FastQC 0.11.3 [27]. Raw sequence reads were aligned to the human reference genome GRCh38 using BWA-MEM v. 0.7.17 [28] and sorted by genomic position using samtools [29]. Picard v. 2.20.2 MarkDuplicates (https://broadinstitute.github.io/picard/) was used to mark duplicate reads, and at the same time merge data from multiple lanes. To identify ”ExAmp^TM^” duplicates (occurring when a library molecule that has formed one cluster is free to go back into solution and create a second cluster, usually close together), we configured MarkDuplicates to use a distance threshold of 2500 pixels. This means that duplicates that are closer than 2500 pixels apart on the images taken by the sequencer get tagged with a Duplicate Type of SQ, to indicate that they are likely to originate from the ExAmp^TM^ process.

The Picard tool DownsampleSam was used to randomly sample reads from each of the libraries, to correct for variations in data yield incurred during pooling and sequencing. Downsampling was performed in two different ways: The first way was to sample 90 million read pairs from each of the input data files. MarkDuplicates was repeated after downsampling. The second way was to select reads corresponding to an average 5.4x genome coverage by the following method. First, we computed the coverage on the full datasets using Picard, and then determined scaling factors for each of the libraries based on the mean coverage. By default, Picard only measures the mean coverage based on non-duplicate reads with a mapping quality of at least 20, base quality Phred score of at least 20, counting overlapping read pairs only once in the overlapping region. Duplicates were removed from the datasets by using samtools to filter on the duplicate flag set by MarkDuplicates. The datasets were then downsampled to the same mean coverage level (as defined above).

Metrics related to alignment and coverage were extracted using both deepTools v. 3.1.3 [30] and Picard. Picard was used to compute the mean coverage and insert size histograms. The tool computeGCBias from deepTools was used to generate Additional Fig. 1B-E, to assess how the observed GC content compares to the GC content expected with a perfectly unbiased method. The nucleotide biases at the start of the reads, in Additional Fig. 1F, were derived from the metrics produced by FastQC.

Short variants were called using GATK v. 4.1.7.0 [31] HaplotypeCaller, and then scored using the GATK tool CNNScoreVariants. The default parameters and model were used. Variant calls were compared to the “confident calls” from the Platinum Genomes [32] version 2017-1.0 dataset, using hap.py v. 0.3.8 (https://github.com/Illumina/hap.py). To condense the SNV and indel calling performance into two single numbers per library, the F_1_ score was computed at a threshold -5 of the GATK machine learning model (CNNScoreVariants) quality score. The threshold was chosen because all the libraries had the peak F_1_ scores around this value, and the precision and recall were at reasonable levels. The score is defined as F_1_=2 / (recall^-1 + precision^-1), and the precision and recall values are taken from the outputs of hap.py. Insertions and deletions of all sizes were included in the indel calling performance. The visualisation of the intersections of variant calls was created using a custom Python script to iterate over the VCF files from hap.py, in combination with UpSet plots [33].

### Statistical analysis

The significance of the difference in the mean insert sizes was computed with Student’s t-test, with unequal variances. The test was applied between all pairs of replicate groups, testing the libraries with 10 ng and 100 ng inputs separately. The hypothesis that the mean coverage depths of Swift2S (10 ng only) and Nextera were different from the other kits was also tested using two t-tests, one for each input concentration value. These tests were applied to the libraries in question, versus all other libraries as a group.

Regression analysis of the coverage distributions was done using the curve_fit function from the SciPy Python library [34]. The number of nucleotides covered at a read depth of k is was modelled as a Poisson probability mass function multiplied with a normalisation constant: f(k; λ, N) = N*(λ^k^ e^-λ^) / (k!). The parameter λ is equal to the expected value of the distribution. The observed number of genomic positions with zero coverage can be written as X_0_ + f(0; λ, N), where X_0_ represents an excess due to regions that cannot be sequenced. The two terms are correlated due to the fixed reference genome size (X_0_ + N = reference size). Accounting for fixed read-counts, as in our experiment (λ =constant/N), we can find the derivative df(0; λ(X_0_), N(X_0_)) / dX_0_ = -e^- λ^(λ-1). Because the value of λ is approximately 5 in our dataset, the correlated changes in f(0) are less than 3 % of the changes in X_0_. We can therefore ignore this correlation and consider the variance in X_0_ only. We then applied the t-test to the observed number of nucleotides with zero coverage, after subtracting the estimated Poisson contributions, to test if the percentage of the genome that cannot be sequenced is significantly different between the Nextera kit and the others.

The linear regression of the insert size versus F_1_ score was done using a linear model (lm) in the R software package.

## Supplementary Information

**Additional file 1: Table S1.** Individual library concentrations.

**Additional file 2: Figure 1**. Fragmentation profiles of libraries as assessed by Tapestation and GC content and bias of reads from different libraries. **Figure 2.** Sequencing data yield and performance of libraries. **Figure 3**. Comparison of genome coverage to the theoretical Poisson distribution. **Figure 4.** Correlation between variant calling performance and insert size. **Figure 5.** Intersections of variant calls.

## Data Availability

The datasets generated and/or analysed during the current study are available in the NCBI SRA repository (https://www.ncbi.nlm.nih.gov/sra) under submission PRJNA769842.
