# Supplementary material for: Optimization of enzymatic fragmentation is crucial to maximize genome coverage: a comparison of library preparation methods for Illumina sequencing
Source: BMC Genomics. 2022 Feb 1;23:92. doi: 10.1186/s12864-022-08316-y (PMC8805253; doi:10.1186/s12864-022-08316-y)
Supplement: Supplementary file 2 — Additional file 2: Figure 1. Fragmentation profiles of libraries as assessed by Tapestation and GC content and bias of reads from different libraries. Figure 2. Sequencing data yield and performance of libraries. Figure 3. Comparison of genome coverage to the theoretical Poisson distribution. Figure 4. Correlation between variant calling performance and insert size. Figure 5. Intersections of variant calls. [file 12864_2022_8316_MOESM2_ESM.pdf]

Additional Figure 1

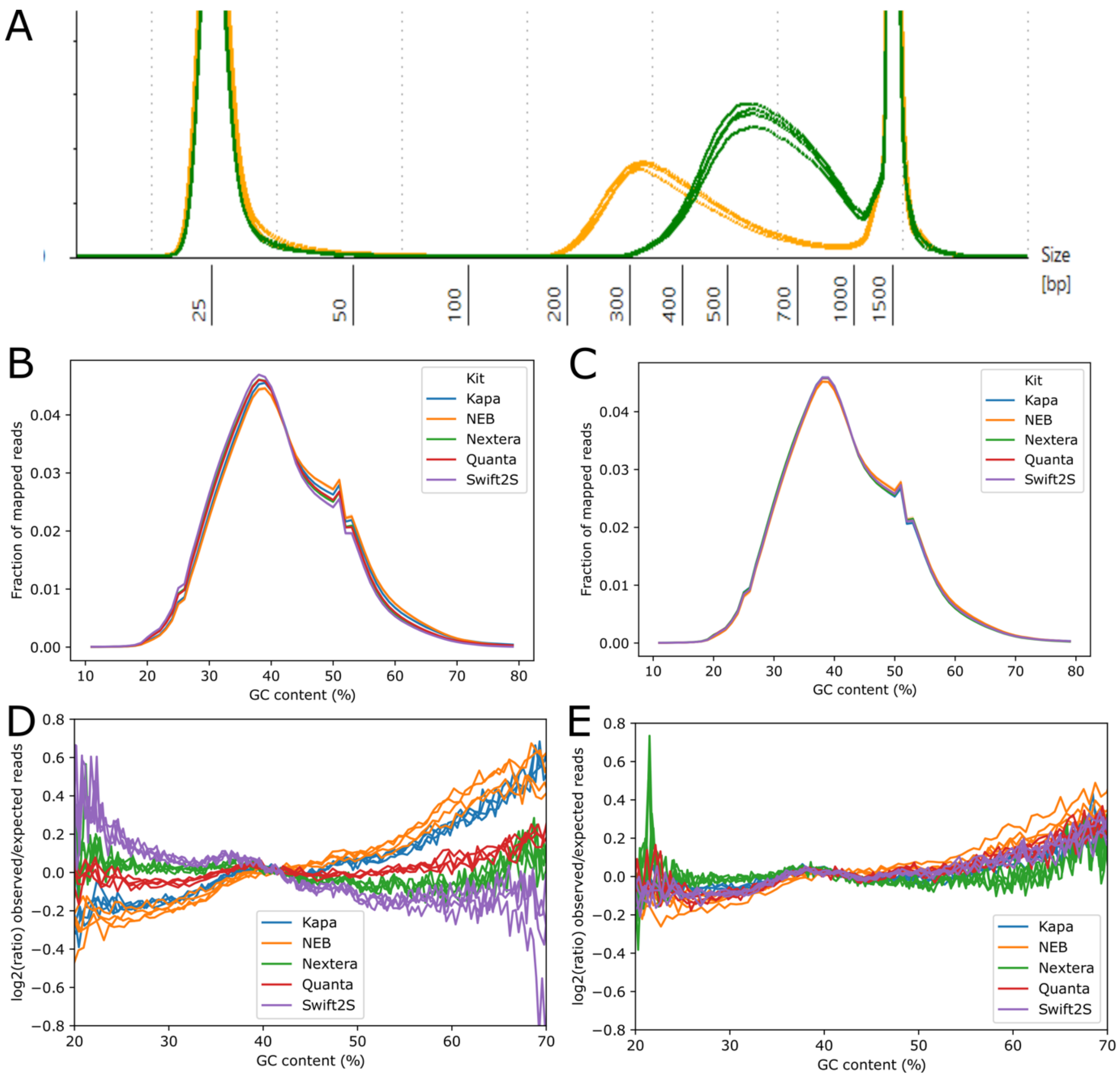

F

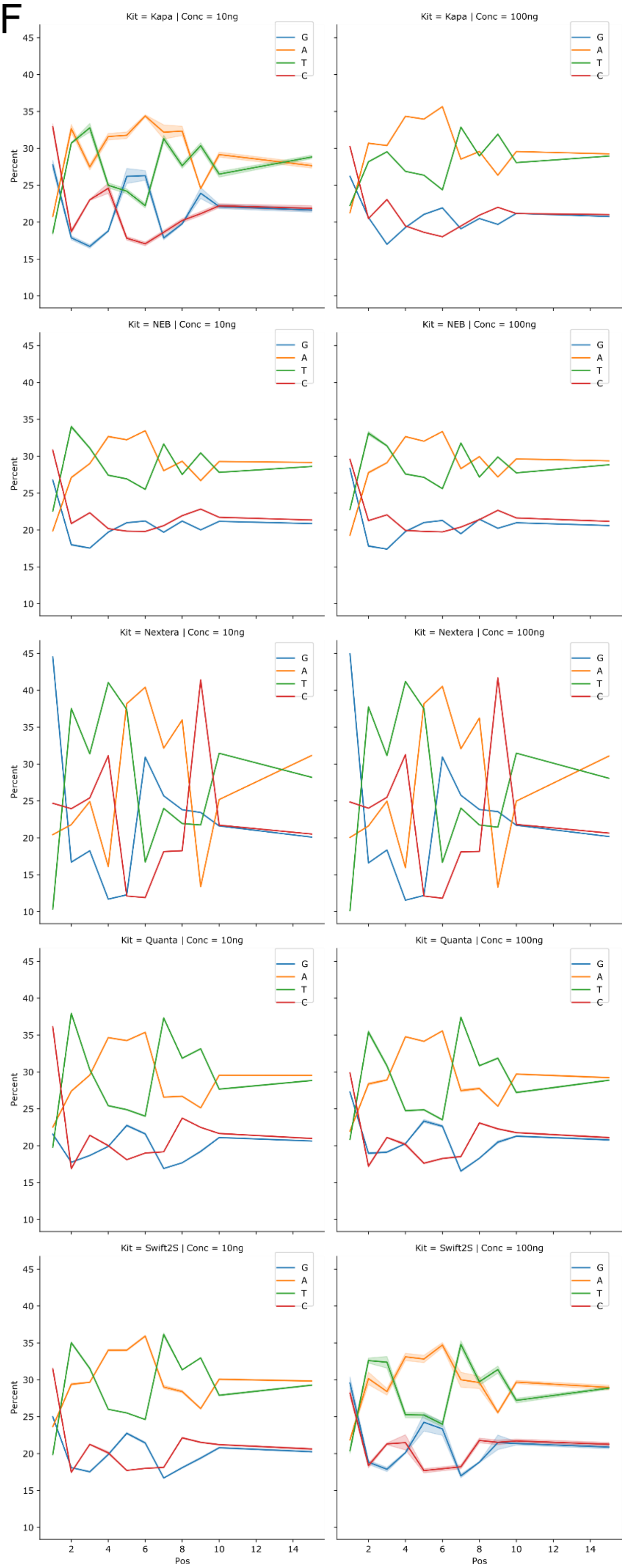

Kit = Kapa | Conc = 10ng
Kit = Kapa | Conc = 100ng
Kit = NEB | Conc = 10ng
Kit = NEB | Conc = 100ng
Kit = Nextera | Conc = 10ng
Kit = Nextera | Conc = 100ng
Kit = Quanta | Conc = 10ng
Kit = Quanta | Conc = 100ng
Kit = Swift2S | Conc = 10ng
Kit = Swift2S | Conc = 100ng
G
A
T
C
Percent
Pos

Additional Figure 1. Fragmentation profiles of libraries as assessed by Tapestation and GC content and bias of reads from different libraries

(**A**) Tapestation D1000 electorphoresis profiles for libraries prepared with 100 ng input DNA including PCR in four technical replicates each with Nextera DNA flex (Illumina) (green) and NEBNext Ultra II FS (NEB) (orange) PCR-free libraries (from Kapa, SparQ and Swift from 100 ng input) were not analysed, as they do not migrate properly due to their forked adapters. (**B, C**) Percentage GC content distribution of the sequencing reads as fraction of the mapped reads or as observed: expected ratio (**D, E**) of the sequencing reads from libraries produced from 10 ng (left panels) and 100 ng (right panels) input DNA. Abbreviations are Nextera DNA flex (Illumina), NEBNext Ultra II FS (NEB), Kapa Hyper Plus (Roche), SparQ DNA library (Quantabio) and Swift2S turbo flex (Swift) kits. (**F**) Base content percentage in the beginning (first 14 bases) of the reads for each library type and DNA input.

Additional Figure 2

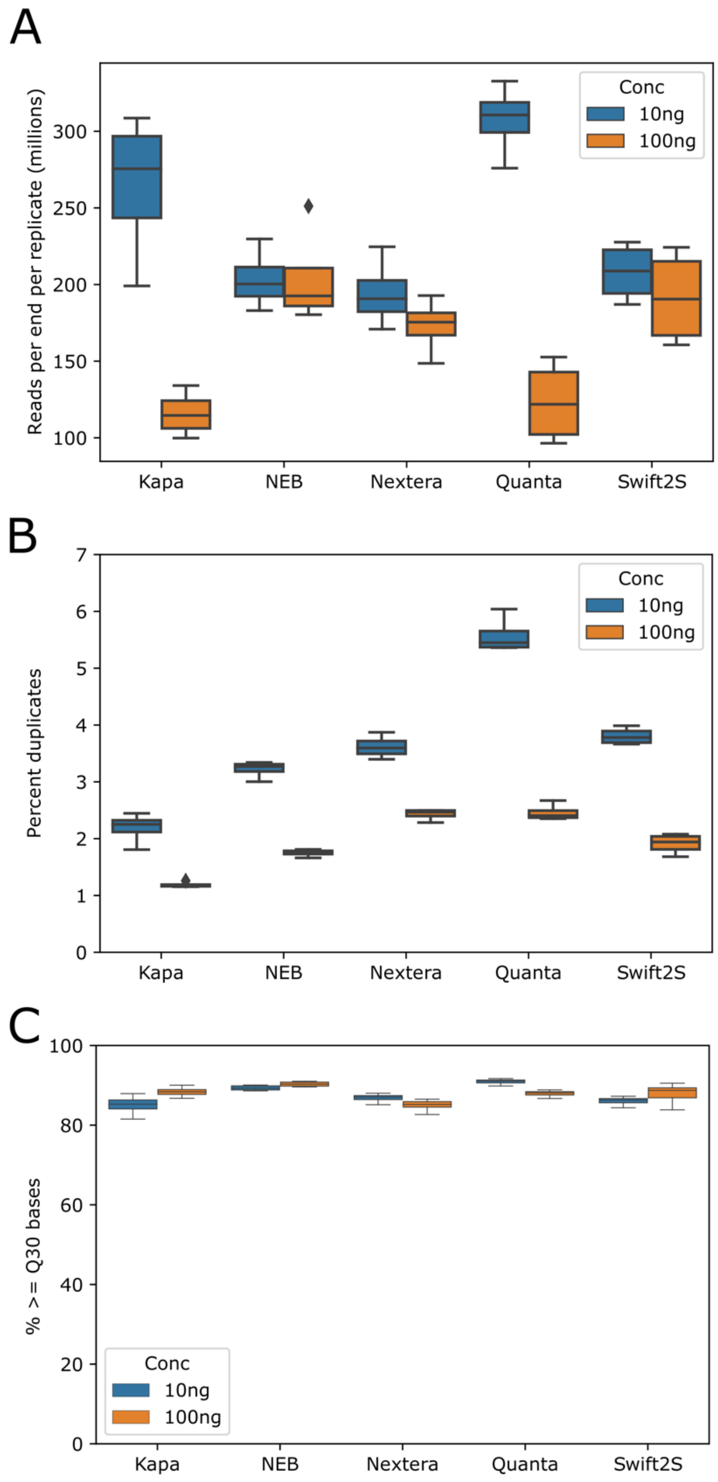

Additional Figure 2. Sequencing data yield and performance of libraries

Libraries were prepared from 10 ng and 100 ng DNA input using Nextera DNA flex (Illumina), NEBNext Ultra II FS (NEB), Kapa Hyper Plus (Roche), SparQ DNA library (Quantabio) kits and Swift2S turbo flex (Swift) kit. (**A**) Sequencing output as total million reads per replicate, (**B**) non-examp duplicates content (%), (**C**) Percentage of bases with Quality scores above Q30.

Additional Figure 3

A

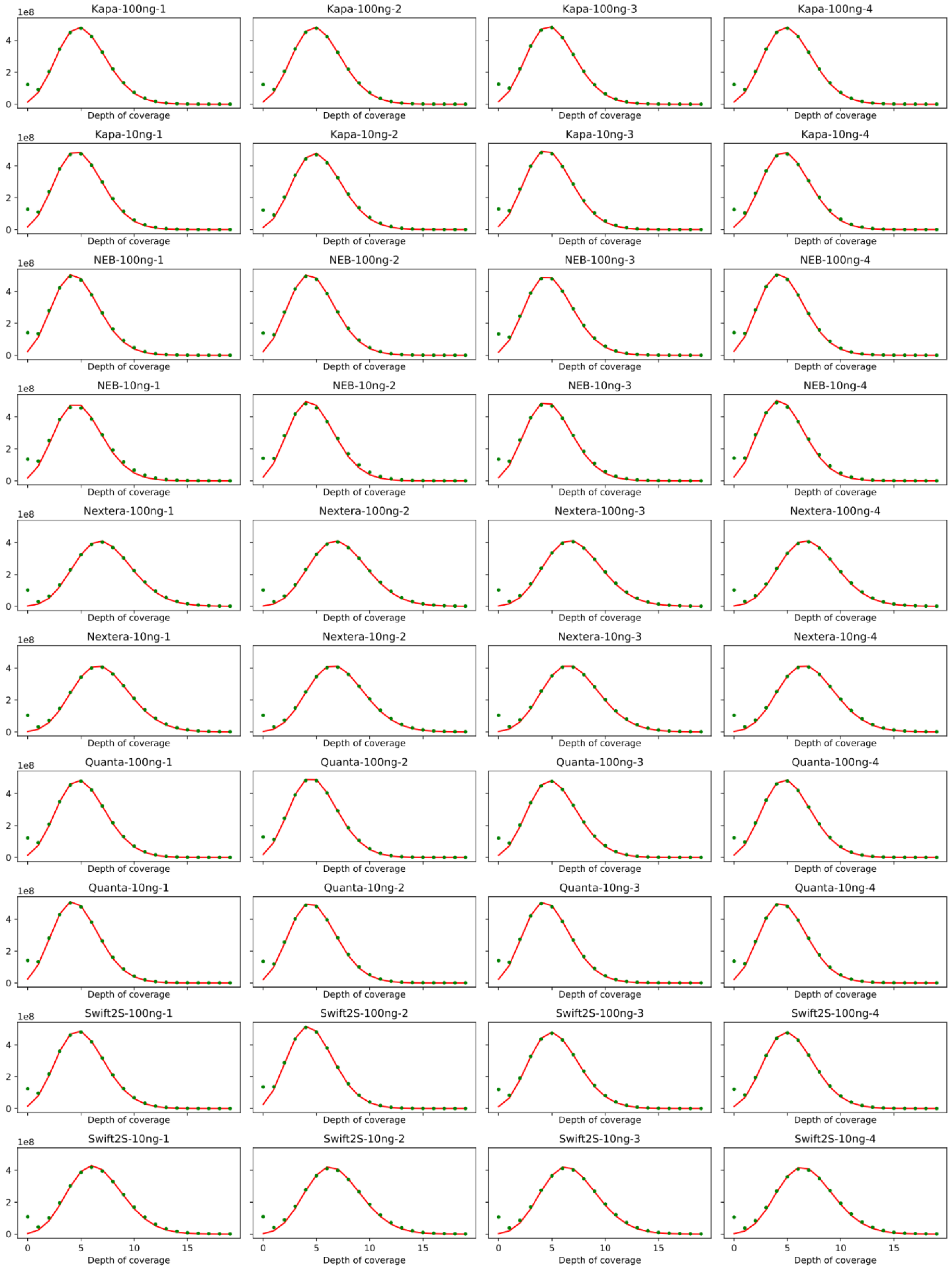

B

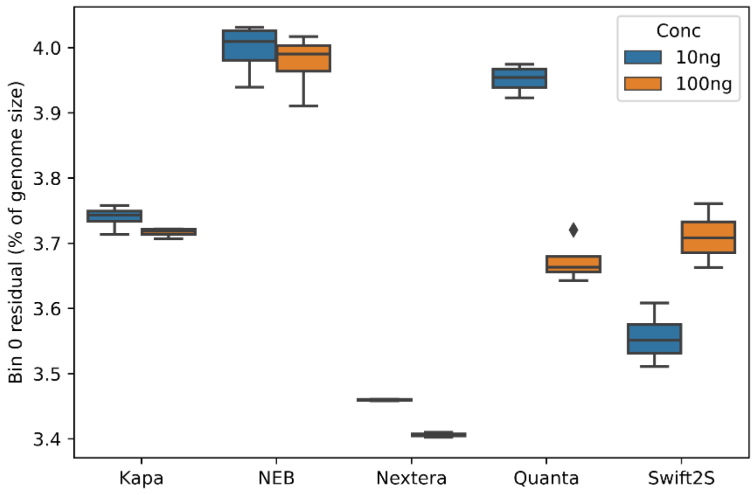

Additional figure 3. Comparison of genome coverage to the theoretical Poisson distribution.

(**A**) Coverage histograms fitted with Poisson distributions in bins of 1-20 X coverage depth. Data points are shown with green dots, and the Poisson distributions with red lines. The fitted functions are also evaluated in bin 0 (i.e. zero coverage). (**B**) The absolute excess (residual) observed at zero coverage, converted to a percentage of the genome size. Error bars indicate variation among the replicates.

Additional Figure 4

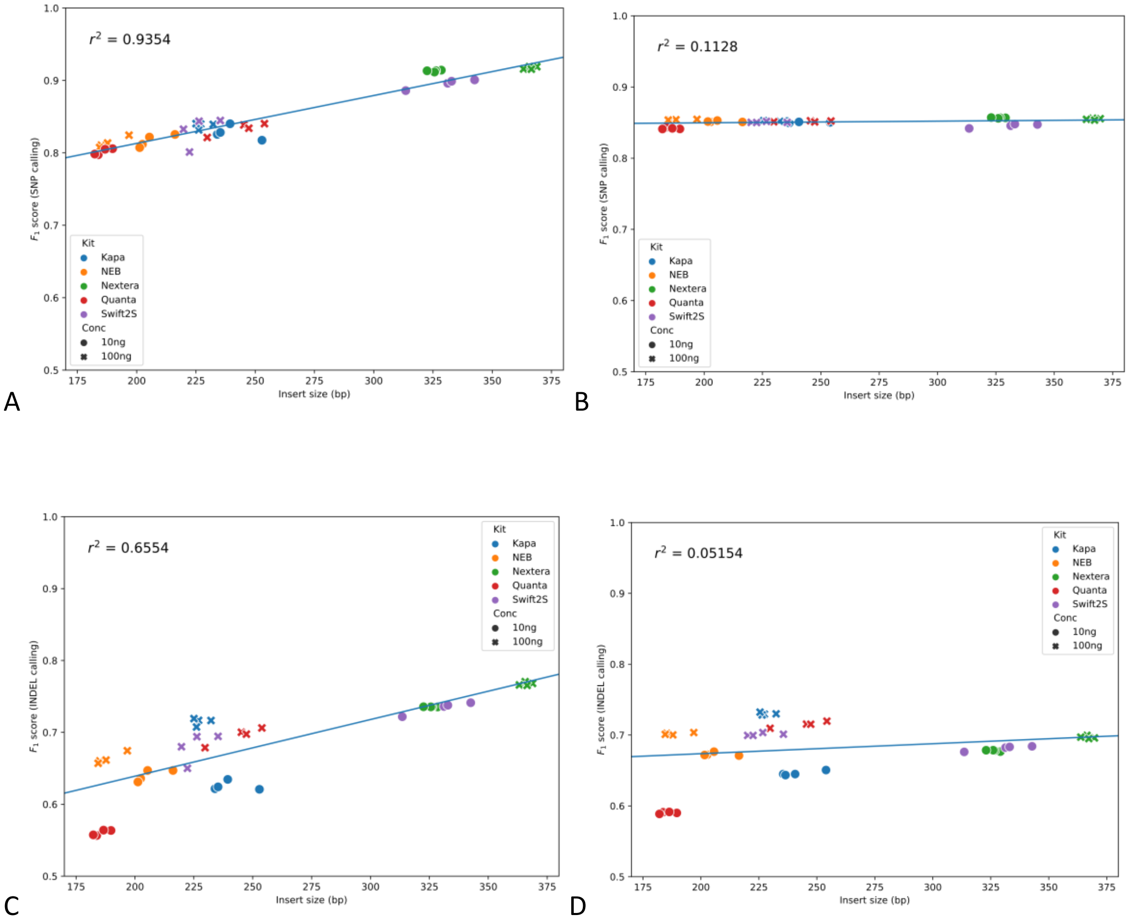

Additional Figure 4. Correlation between variant calling performance and insert size.

The F_1 scores are fitted with straight lines as functions of insert size. Figures **(A, B)** show the SNP calling performance and **(C,D)** show the INDEL calling performance. On the left **(A,C)** the data are normalized to a the same number of reads, and on the right **(B,D)** to a fixed average depth of coverage.

Additional Figure 5

A

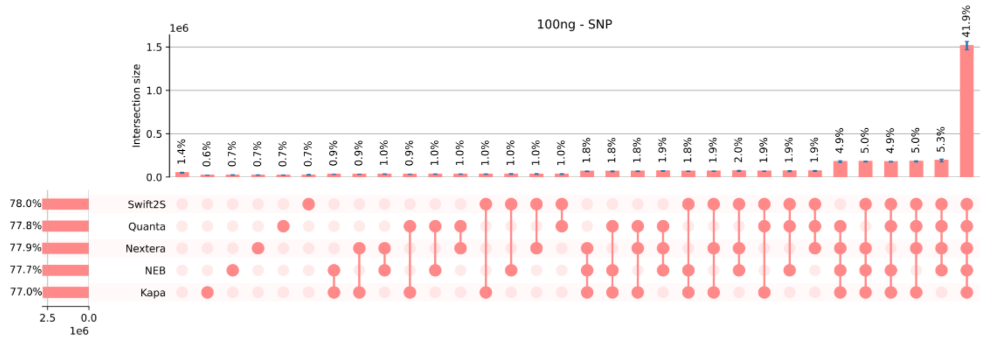

B

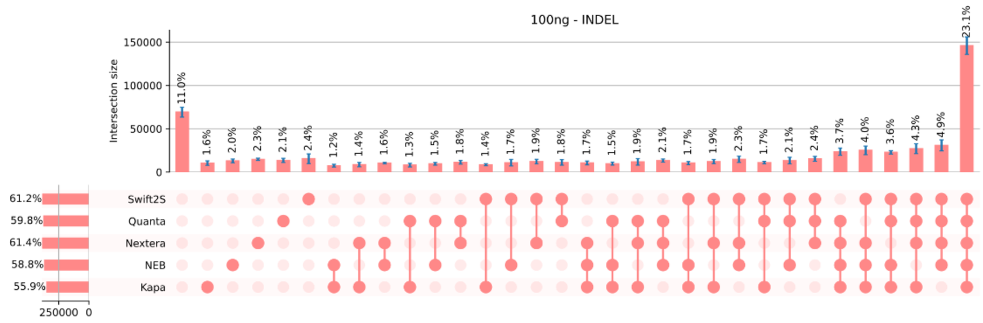

C

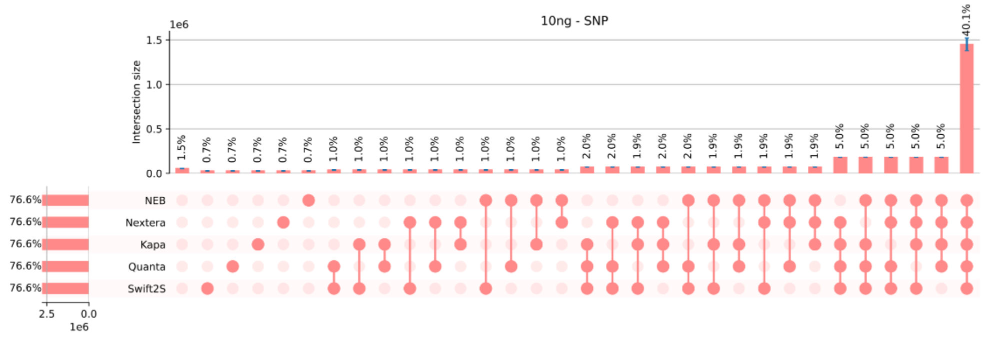

D

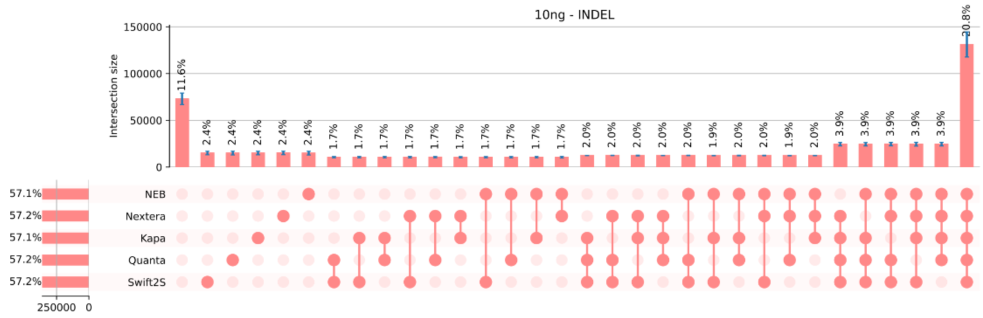

Additional Figure 5. Intersections of variant calls.

The plots show overlaps of the true positive variant calls among the kits, based on coverage-normalised (5.4x) data. The central barplot shows the number of variants in each intersection, and the percentage of the whole dataset. The overlaps are computed four times, for each set of replicates, and the error bars indicate the spread among these data. The first two plots (A-B) show the 100 ng input libraries, and the next plots (C-D) show the 10 ng input libraries.
